# Beyond Legacy PFAS: Dominant Role of Ultrashort-Chain and Emerging PFAS in the Elbe River−Sea Continuum

**DOI:** 10.1021/acsenvironau.5c00219

**Published:** 2026-03-18

**Authors:** Anne Röhrig, Nico Grasse, Martin Krauss, Dana Bücher, Werner Brack, Norbert Kamjunke, Anna Matousu, Tina Sanders, Ingeborg Bussmann, Eric P. Achterberg, Thorsten Reemtsma, Qiuguo Fu

**Affiliations:** † Department of Environmental Analytical Chemistry, 28342 Helmholtz-Centre for Environmental Research − UFZ, Permoserstrasse 15, Leipzig 04318, Germany; ‡ Department of Exposure Science, 28342 Helmholtz-Centre for Environmental Research − UFZ, Permoserstrasse 15, Leipzig 04318, Germany; § Institute for Analytical Chemistry, University of Leipzig, Linnestrasse 3, Leipzig 04103, Germany; ∥ Department of Evolutionary Ecology and Environmental Toxicology, Faculty of Biological Sciences, Goethe University Frankfurt, Max-von-Laue-Strasse. 13, Frankfurt am Main 60438, Germany; ⊥ Department of River Ecology, Helmholtz Centre for Environmental Research − UFZ, Magdeburg 39114, Germany; # 90801 Biology Centre, Czech Academy of Sciences, Institute of Hydrobiology, Ceské Budejovice 370 05, Czech Republic; ∇ Institute of Carbon Cycles, Helmholtz Centre Hereon, Geesthacht 21502, Germany; ○ Department of Shelf Sea System Ecology, Alfred-Wegener-Institut, Helmholtz Zentrum für Polar- und Meeresforschung, Helgoland 27498, Germany; ◆ 28402 GEOMAR, Helmholtz Centre for Ocean Research Kiel, Kiel 24148, Germany

**Keywords:** persistent chemicals, river−sea systems, PFAS, environmental monitoring, RPLC-MS/MS, SFC-MS/MS

## Abstract

Per- and polyfluoroalkyl substances (PFAS) are highly persistent contaminants. Current regulatory monitoring frameworks cover only a narrow set of regulated long- and short-chain PFAS, while ultrashort-chain compounds such as trifluoroacetic acid (TFA) remain largely unaddressed despite their widespread occurrence. Here, we present regional sources and tributary inputs of diverse PFAS (*n* = 83), including ultrashort-, short-, and long-chain PFAS as well as PFAS precursors along the 1,200 km Elbe River−North Sea continuum. Based on 129 samples from the Elbe, its tributaries, and wastewater treatment plants (WWTPs), we show that short- and long-chain PFAS concentrations in the Elbe were generally below 30 ng/L, but 1,653 ± 377 ng/L when including TFA. Major tributaries such as the Saale, Vltava, and Mulde delivered PFAS mass loads of several thousand g/day (2,340; 6,080; and 1,700 g/day, respectively, when including TFA) and together accounted for a total discharge of 160 m^3^ s^−1^, corresponding to approximately 67% of the Elbe’s water flux at Geesthacht (240 m^3^/s), which represents the downstream reference point for mass balance calculations prior to tidal influence. This suggests source-enriched inputs rather than simple dilution by tributary inflows, given the disproportionate increase in PFAS mass loads relative to discharge. WWTP effluents from Dresden and Pardubice added up to 185 g/day, mainly shaped by short-chain PFAS. The detection of Capstone B (CDPOS) and Bis­(trifluoromethylsulfonyl)­imide (NTf2), non-routinely monitored industrial PFAS, further highlights overlooked sources in the area of the Saale-Elbe confluence. Inclusion of TFA increased the median PFAS load in the Elbe River by nearly 50-fold (up to 39,958 g/day), demonstrating the dominant role of ultrashort-chain PFAS currently absent from regulatory monitoring. By linking source apportionment with river-to-sea transport, our study highlights critical gaps in existing monitoring frameworks and provides a transferable methodology for more comprehensive PFAS assessment and regulation in large river systems.

## Graphical abstract

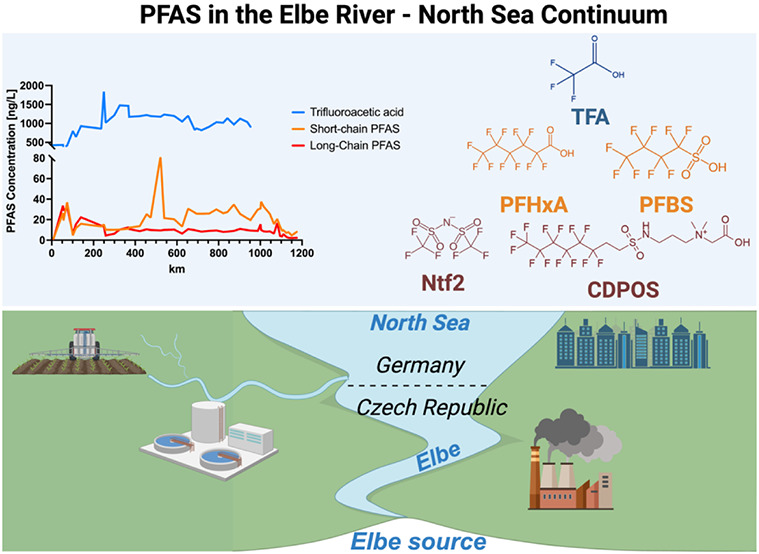

## 1 Introduction

Per- and polyfluoroalkyl substances (PFAS) are a large group of synthetic chemicals (*n* > 4700 ^1,2^ ) and have emerged as a critical concern due to their widespread use, persistence in the environment, and potential adverse effects on human health and ecosystems.^3–5^ With diverse structures and physicochemical properties, PFAS are often categorized based on their carbon chain length. In this study, we apply an operational classification of ultrashort (C1−C3), short (C4−C7), and long (>C7) chain PFAS for comparative analysis, representing a simplified form of commonly used regulatory and review definitions that distinguish between perfluoroalkyl carboxylates and sulfonates.^1^ While all PFAS exhibit environmental persistence, their environmental behavior and toxicological profiles differ substantially. Long-chain PFAS, such as PFOA and PFOS, exhibit high affinity for proteins^6^ and bioaccumulation potential,^5,7^ while short and ultrashort-chain PFAS are less bioaccumulative but more mobile and harder to control and remediate.^8^

PFAS contamination is now recognized as ubiquitous, with particularly high relevance in surface waters that integrate inputs from diffuse and point sources.^9–11^ Rivers play a central role in the environmental transport of PFAS, linking inland emissions to downstream aquatic and marine ecosystems.^11–14^ The Elbe River stretches over more than 1,000 km from its headwaters in the Czech Republic through Germany to the North Sea, and it is a major European waterway that is affected by a legacy of industrial activity, dense urbanization, and wastewater discharge.^15^ Previous studies have reported PFAS concentrations in Elbe waters,^16^ sediments,^9,17^ and biota,^18^ with known hotspots in Czech and German regions.

Despite increasing monitoring efforts, current knowledge of PFAS dynamics in river−sea systems remains fragmented. Many studies focus on a limited set of legacy compounds, often excluding ultrashort-chain PFAS (e.g., trifluoroacetic acid (TFA)) and PFAS precursors due to analytical challenges. PFAS precursors, such as fluorotelomer- and sulfonamide-based compounds, can undergo biotic and abiotic transformation during wastewater treatment as well as in natural aquatic and sedimentary environments, leading to the formation of terminal perfluoroalkyl acids (PFAAs) including PFOA and PFOS. These processes involve oxidative, microbial, and photochemical pathways and may result in increased loads of stable and persistent PFAAs relative to their precursors.^19–21^

In highly dynamic environments such as estuaries, where salinity gradients, mixing, suspended particles, and sedimentation interact, the combined effects of upstream transformation and *in situ* partitioning and remobilization processes on PFAS mixture profiles remain poorly constrained.^22,23^ Furthermore, the contribution of tributaries as secondary, catchment-scale inputs that integrate multiple diffuse and point sources, and of wastewater treatment plants (WWTPs) as discrete point sources of both parent PFAS and degradable precursors, is not well resolved on a river-wide scale of the Elbe. In addition, emerging PFAS such as Capstone B (CDPOS) remain insufficiently characterized, with limited data available on their environmental occurrence, fate, toxicity, and emission sources. These compounds are not yet routinely included in monitoring programs, which constrains current assessments of their distribution and relevance in aquatic systems.

Although German federal states have conducted regional PFAS monitoring, these data sets often differ in scope, frequency, and analytical coverage; for example, the “SumPFAS” project commissioned by the UBA compiled monitoring data on PFAS in surface waters from all states, including the Elbe, Rhine, Donau, Saale, and Mulde catchments. Consequently, while useful insights exist at local scales, the full contribution of tributaries and WWTPs to PFAS loads in the Elbe River basin remains insufficiently resolved.

To address these gaps, comprehensive data sets combining compound-specific quantitative data, high spatial resolution, and multivariate analysis are needed to link PFAS profiles to sources and transport processes and to inform targeted regulatory and mitigation strategies. In this study, we quantified a broad suite of diverse PFAS (n = 83), including ultrashort-, short-, and long-chain PFAS as well as rarely monitored compounds such as CDPOS and NTf2, across river, tributaries, and WWTP effluents. Using hierarchical clustering, we resolved spatial fingerprints and potential source contributions. Our study aims to (i) refine the understanding of PFAS distribution and transport along the Elbe continuum, (ii) identify potential source inputs, particularly linked to tributaries and WWTPs, and (iii) assess the role of ultrashort-chain and emerging PFAS currently overlooked in monitoring frameworks. By providing a river-wide, compound-resolved assessment of PFAS concentrations and mass loads, this work supports the development of source-specific mitigation strategies and informs efforts to expand regulatory monitoring beyond legacy PFAS.

## 2 Materials and Methods

### 2.1 Standards and Reagents

For quantification of PFAS, reference standards and isotopically labeled standards were obtained from Wellington Laboratories Inc. (Canada). In total, 83 target PFAS were quantified using 28 isotopically labeled standards. SI_A: Tables S1−2 provide details about the chemicals and internal standards.

### 2.2 Sampling

The samples analyzed in this study were collected during the MOSES (Modular Observation Solutions for Earth Systems) campaign performed during low discharge conditions in summer of 2023.^24^ A total of 129 water samples were collected from 84 sites along the Elbe River to the North Sea between June 27 and September 14, 2023, including influent and effluent samples from 11 WWTPs, 72 river samples from 33 river sites along the Elbe River (without WWTPs and tributaries)—between Špindlerův Mlýn (8 km) and Geesthacht (953 km), 16 tributaries, as well as seawater samples from ten tidal sites, and nine German Bight samples (Figure 1). For each sample, 500 mL of water was collected in polypropylene bottles. The locations of the WWTPs and tributary confluences are shown on Figure S4 in SI_A. In the Czech section, grab samples were taken due to frequent weirs preventing Lagrangian sampling i.e., tracking the same water parcel downstream. From the Czech-German border to Geesthacht, a Lagrangian sampling according to flow velocity was applied using the research vessel (RV) Albis. Each river site of the German freshwater Elbe was sampled laterally (center and both banks). WWTP influent and effluent samples were collected as 24 h composites using autosamplers, time-synchronized at each WWTP. Although sampling was aligned, the actual hydraulic retention time (HRT) may differ from 24 h. Sampling of the tidal Elbe was performed 6 weeks after the freshwater river which is the mean residence time in the confluence at low discharge, and a quasi-steady-state approach was used, sampling upstream during ebb tide with RV Ludwig Prandtl in 2 days. Coastal samples were taken with RVs Littorina and Mya II using a CTD rosette. Samples were stored at 4 °C and frozen at −20 °C the same day. Field blanks accompanied the campaign and were prepared by filling sample bottles on board with LC−MS grade water from a storage bottle. Due to the size and design of the sampling devices, blanks could not be processed directly through the samplers. All detailed sampling locations are provided in SI_B: Table S3.

**Figure 1:**
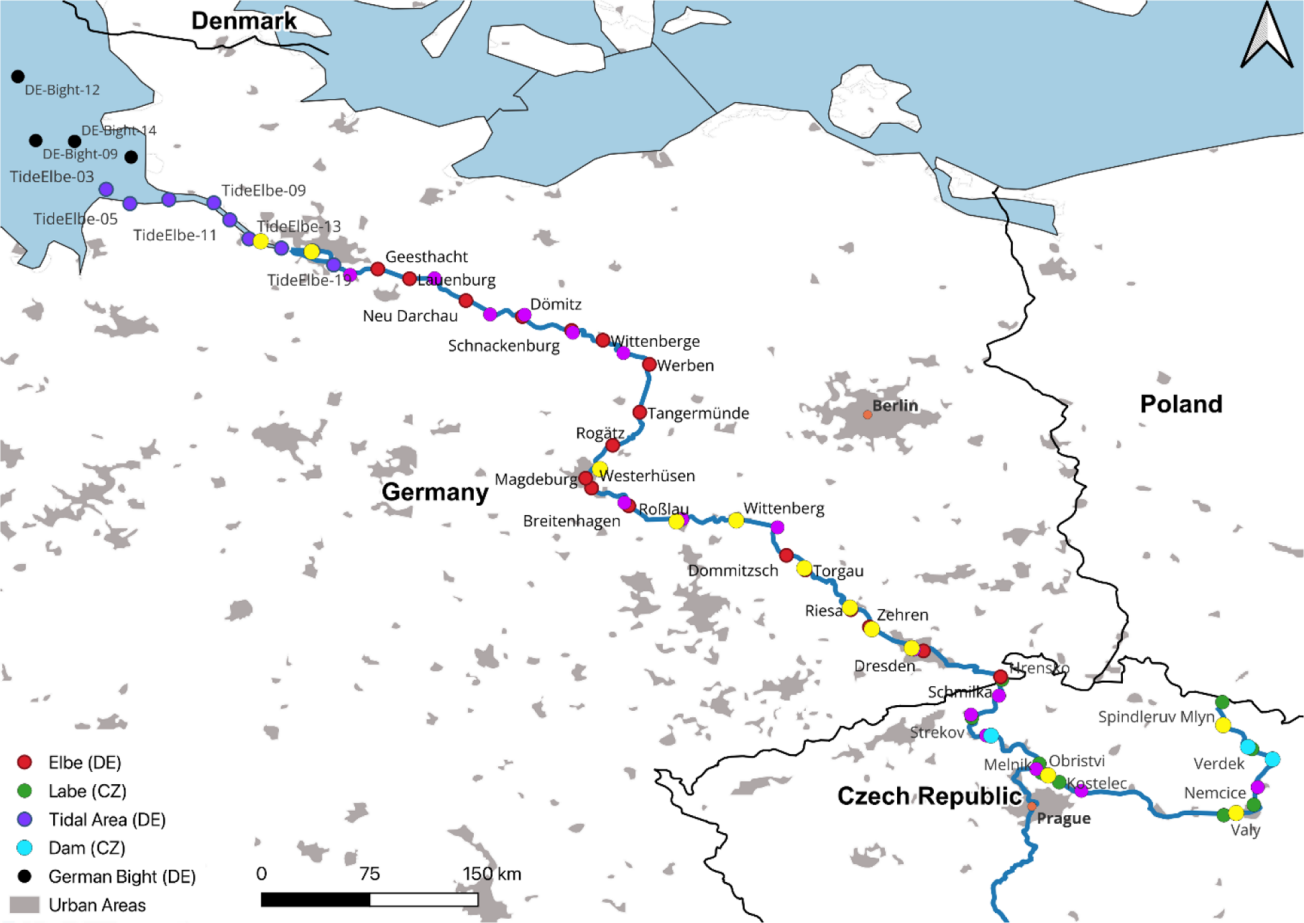
**Overview of sampling locations along the Elbe River to the North Sea**. Samples were collected in 2023 in the Czech (CZ) River Elbe, German (DE) River Elbe, the Elbe confluence and the North Sea (German Bight and Elbe confluence) and WWTPs and tributaries. Urban areas are indicated as gray areas. Sites close to dams in the Czech Republic are indicated as light blue dots. Other areas not specified in the map include agricultural land, forests, and similar land-use types.

### 2.3 Sample Preparation

The extraction method was adapted from Meier et al. with modifications: the sample volume was reduced from 40 to 25 mL and further no freezing-round-bar was used during lyophilization enrichment.^25^ For each sample, 25 mL of water (river water, WWTP influent, and WWTP effluent) was weighed into two precleaned 50 mL FALCON tubes (Corning Inc.). One aliquot was used for the analysis of short- and long-chain PFAS by ultraperformance liquid chromatography−tandem mass spectrometry (UPLC−MS/MS). The second aliquot was used for the analysis of ultrashort-chain PFAS (e.g., trifluoroacetic acid, TFA) by supercritical fluid chromatography−tandem mass spectrometry (SFC−MS/MS), which provides improved retention and robust quantification for highly polar, low-molecular-weight PFAS compared to conventional reversed-phase LC methods.^26,27^

The first aliquot was spiked with an absolute amount of 500 pg of each isotopically labeled standard. The aliquots were frozen overnight and freeze-dried within 48 h. Residues were rinsed down in a two-stage procedure with acetonitrile/water azeotrope, which was evaporated under a nitrogen stream. Residues were dissolved in 250 μL of final solvent, centrifuged at −5 °C and transferred into polypropylene HPLC vials. The final solvent for PFAS analysis via UPLC was methanol/water (1:1), for ultrashort-chain PFAS analysis via SFC, it was acetonitrile/water (9:1).

For tidal and German Bight water samples, a solid-phase-extraction (SPE) method was used for enrichment and cleanup due to the high salt content in these water samples. The sample pH was adjusted to 5−6 using formic acid before using SPE. After conditioning the SPE (Oasis PFAS WAX/GCB (Part No 186011110; WAX 200 mg 60 μm/GCB 50 mg) cartridges with basic methanol (0.3% NH_4_OH), methanol and ultrapure water (Milli-Q, Millipore) the cartridges were loaded with a mixture of 100 mL of sample and 300 mL of ultrapure water each (flow rate 4 mL/min). After sample load, cartridges were washed with ultrapure water, dried by suction and eluted with basic methanol in two elution steps. The eluate was blown down to 1 mL using a N_2_ evaporator.

### 2.4 PFAS Analysis

All reported concentrations were blank-corrected by subtracting the concentrations measured in the field and process blanks from those observed in the samples. The PFAS analysis was carried out by reversed-phase (RP)-UPLC-MS/MS. For analysis of (ultra)­short-chained PFAS, an additional measurement was carried out by SFC-MS/MS. Both systems are from Waters Corporation. Data acquisition, integration and quantitation were performed using MassLynx software version 4.2. Sea water samples from the tidal area and the German Bight were not injected into the SFC-MS/MS to prevent high salt load in the instrument. Method performance was provided in the Results and Discussion below, further validation data, including method detection and quantification limits, recoveries, and matrix effects, are provided in SI_A: Tables S1, S4 and S5.

#### 2.4.1 RPLC-MS/MS

RPLC-MS/MS was performed using a TQS system (Waters, USA) and the water/methanol extracts. UPLC separation was achieved using an ACQUITY UPLC BEH Shield RP 18 column with 1.7 μm, 2.1 × 50 mm (Waters) with gradient elution at 350 μL/min flow rate and an injection volume of 5 μL. Details on the gradient and eluent composition are given in the Supporting Information SI_B: Table S1. To minimize possible PFAS contaminations from eluents or instrument parts, an isolator column (Waters Corp Part. 186004476) was installed upstream of the autosampler. Electron spray ionization (ESI) source parameters were optimized as follows: capillary voltage 1 kV, source temperature 150 °C, desolvation temperature 350 °C, cone gas flow 150 L/h and desolvation gas flow 600 L/h.

#### 2.4.2 SFC-MS/MS

SFC was performed using a TQXS system (Waters, USA) for analyzing ACN/water extracts. SFC separation was achieved using a Torus Diol Column, 130Å, 1.7 μm, 3 mm × 100 mm (Waters Corp. PartNo. 186007611) for negative mode acquisition. The gradient elution at a 1.2 mL/min flow rate and an injection volume of 5 μL used the gradient and eluent composition given in SI_B: Table S2. ESI source parameters were optimized as follows: capillary voltage 2 kV, cone voltage 20 V, source temperature 140 °C, desolvation temperature 550 °C, cone gas flow 150 L/h and desolvation gas flow 950 L/h.

### 2.5 Hierarchical Clustering and Principal Component Analysis

To identify spatial patterns in PFAS contamination along the Elbe River, hierarchical cluster analysis and principal component analysis (PCA) were conducted on log-transformed and Z-scaled concentration data (maximum per site) (SI_B: Figure S4). Sites and compounds were clustered using Ward’s method and Euclidean distance, with the optimal number of clusters determined via silhouette width. Heatmaps and principle component analysis (PCA) biplots (including 95% confidence ellipses) visualized grouping patterns and explained variance. Sampling sites were mapped in QGIS using georeferenced coordinates. Detailed information on data preprocessing, clustering, PCA implementation, and GIS settings is provided in the SI_B: Sections 2 and 5.

### 2.6 Hydrological and Auxiliary Data

#### 2.6.1 Discharge Values, Population Equivalents, and Water Chemistry

Discharge values and population equivalents (PE) for tributaries and WWTPs were compiled from local gauge data, official water authority reports, and EU databases (SI_A: Table S6). Discharge data were retrieved or interpolated from site-specific measurements at the nearest hydrological stations (e.g., Czech Hydrometeorological Institute, German Bundesanstalt für Gewässerkunde) and are provided in SI_A: Table S6. For sampling sites in the Binnenelbe section, the total river discharge was partitioned laterally by assigning 25% of the discharge to the left channel, 50% to the midchannel, and 25% to the right channel, consistent with the hydrodynamic assumptions used in the accompanying modeling framework. Population equivalents were derived primarily from the European Waterbase UWWTD 2022 data set^28^ and supplemented by national reporting data where necessary.

Additionally, basic water chemistry parameters (e.g., temperature, conductivity, O_2_, Fe, Mn, CO_2_, CH_4_, N_2_O) were collected in parallel at selected river sites and are documented in the PANGAEA repository under data set PANGAEA:963359 ^29^ and provided in SI_A: Table S7.

#### 2.6.2 Calculation of PFAS Mass Loads

PFAS mass loads (L, g/day) were calculated from measured concentrations (c, ng/L) and river discharge (Q, m^3^/sec) according to

1 L=c×Q×0.0864

The factor 0.0864 converts units from ng/L and m^3^/sec to g/day. Uncertainty in mass load estimates arises from both analytical variability in concentration measurements and hydrological uncertainty in discharge assignment, particularly for estimated WWTP and tributary flows. Accordingly, mass loads are primarily interpreted in a comparative, relative framework rather than as exact absolute values.

### 2.7 Risk Quotient (RQ) Analysis

A Risk quotient (RQ) is calculated by comparing a measured environmental concentration (SI_A: Table S8) with the predicted no-effect concentrations (PNECs) which adverse effects are not expected. PNECs data of PFASs for freshwater are derived from the NORMAN Ecotoxicology Database (https://www.norman-network.com/nds/ecotox/lowestPnecsIndex.php). All PNECs and RQ data are provided in SI_A: Table S9 and S10.

2 $$\mathrm{RQ}=\frac{\mathrm{Measured Environmental Concentration}}{\mathrm{PNEC}}$$

RQ < 0.1 indicates low risk; 0.1 ≤ RQ < 1 indicates medium/moderate risk; RQ ≥ 1 indicates potential high risk.

## 3 Results and Discussion

### 3.1 Method Performance

The analytical method showed robust performance for quantifying a diverse suite of PFAS across the Elbe River−North Sea continuum. Median relative recoveries (±standard deviation, SD) were 80 ± 33% for river water, 76 ± 32% for WWTP effluents, 86 ± 41% for WWTP influents, 103 ± 47% for tidal area samples, and 102 ± 42% for German Bight samples (SI_A: Table S4), indicating generally acceptable performance but increased variability in wastewater and coastal matrices.

Method detection limits (MDLs) and method quantification limits (MQLs) ranged from 0.03 to 3.33 ng/L and from 0.08 to 10.0 ng/L, respectively (SI_A: Table S1), which is comparable to values reported for targeted PFAS methods in aqueous matrices^19,27,30^ and sufficient to resolve riverine and tributary concentration gradients observed in this study.

Method precision (RSD, n = 3) was typically ≤ 20% for most target PFAS in river and coastal matrices, whereas higher variability (often >30%) was observed for highly polar PFAS in WWTP influent and effluent samples, reflecting stronger and more heterogeneous matrix effects in wastewater (SI_A: Table S5).

Several compounds exhibited low recoveries (<10%) and/or pronounced matrix effects, including TFA, trifluoromethanesulfonic acid (TFMSA), nafion, perfluorododecanesulfonic acid (PFDoS), perfluorotridecanesulfonic acid (PFTrDS), long-chain compounds (e.g., dodecafluorosuberic acid (PFdiCA), perfluorohexadecanoic acid (PFHxDA), perfluorooctadecanoic acid (PFODA), and certain polyfluorinated phosphate esters (SI_A: Table S4). For TFA, absolute recoveries were 61% in river water, 225% in WWTP effluents, and 5.3% in WWTP influents, indicating strong and variable signal enhancement and suppression. Accordingly, TFA concentrations in wastewater are considered semiquantitative, whereas TFA data in riverine are regarded as more robust.

Overall, while elevated uncertainty applies to highly polar and low-recovery compounds in complex matrices, the consistent spatial patterns and longitudinal trends reported across the Elbe continuum remain robust for comparative and source-oriented interpretation, as they are based on compound-specific internal standardization and performance evaluation.

### 3.2 Spatial Distribution of PFAS along the Elbe River to the North Sea

For quantification, three lateral subsamples were collected at each river site (left bank, midchannel, and right bank), and the median of the three measured concentrations was used to capture representative PFAS levels while minimizing the influence of local heterogeneity and outliers. The following sections present both qualitative and quantitative assessments to establish a comprehensive PFAS profile of the Elbe River to the North Sea. Sampling site positions along the Elbe are reported as river kilometers (km), denoting the distance from the river source in the Špindlerův Mlýn (CZ). Details on the quantitative results are provided in SI_A: Table S8.

#### 3.2.1 TFA Dominates PFAS Profile in the Elbe River

A total of 38 PFAS − including 2 ultrashort-chain, 21 short-chain, and 15 long-chain PFAS − were detected and quantified at 52 sampling sites along the Elbe River, ranging from its headwaters in the Czech Republic to the confluence and German Bight in the North Sea (excluding WWTPs and tributaries). Across all samples, the median concentration of short-chain (C4−C6) and long-chain (>C7) PFAS was 32 ng/L, with values ranging from 4.2 ng/L in Špindlerův Mlýn (8 km) to 91.4 ng/L in Torgau (522 km) (SI_B: Figure S1). Detected ultrashort-chain (<C4) PFAS included TFMSA (8.8 ± 6.0 ng/L) and TFA (1653 ± 377 ng/L).

Because concentrations alone do not reflect the scale of environmental emissions, we calculated PFAS loads (g/day) by combining measured concentrations with daily river discharge (Figure 2A). This provides a quantitative basis to evaluate potential source contributions and quantify river-to-sea fluxes. Among all detected PFAS, TFA accounted for more than 97% of the total PFAS load in the Elbe system. Loads increased from 56 g/day in Špindlerův Mlýn (CZ, 8 km) to 21,718 g/day in Hrensko (CZ, 367 km), closely tracking the rise in river discharge from 0.93 to 104 m^3^/s as the river integrates major tributary inflows along the Czech section. In Germany, PFAS loads ranged between 12,972 g/day (Dresden, 422 km) and 39,958 g/day (Neu Dachau, 903 km). TFA is widely reported as a ubiquitous and persistent contaminant in surface waters, which complicates attribution to specific point or catchment-scale sources.^31^ In the Elbe system, the spatial patterns observed in this study do not allow for a unique source assignment. However, we hypothesize several plausible input pathways, including municipal wastewater effluents,^31^ atmospheric deposition of degradation products from fluorinated refrigerants and pesticides,^32^ and transformation of precursor compounds during wastewater treatment,^31,33^ while industrial emissions such as polymer thermolysis^34^ are considered potential but less well constrained sources. Unlike legacy PFAS, TFA is highly mobile and not retained by soils or sediments, which explains its progressive accumulation along the river and its correlation with discharge (Figure 2A).

Our results highlight its overwhelming contribution in the Elbe and underline the need to systematically assess TFA in other European rivers. For instance, the TFA concentrations in the Elbe river closely match those in the Rhine and Main rivers, where values of around 1400 ng/L have been reported in Düsseldorf and ranged between 1,000 and 1,400 ng/L in the Main river.^31^ This agreement suggests that TFA represents an ubiquitous background contaminant in large European river systems, reflecting its high persistence and mobility rather than isolated local sources. The decision to include or exclude TFA fundamentally alters the interpretation of PFAS pollution in large river systems, emphasizing its critical role for designing future monitoring and regulatory strategies.

**Figure 2:**
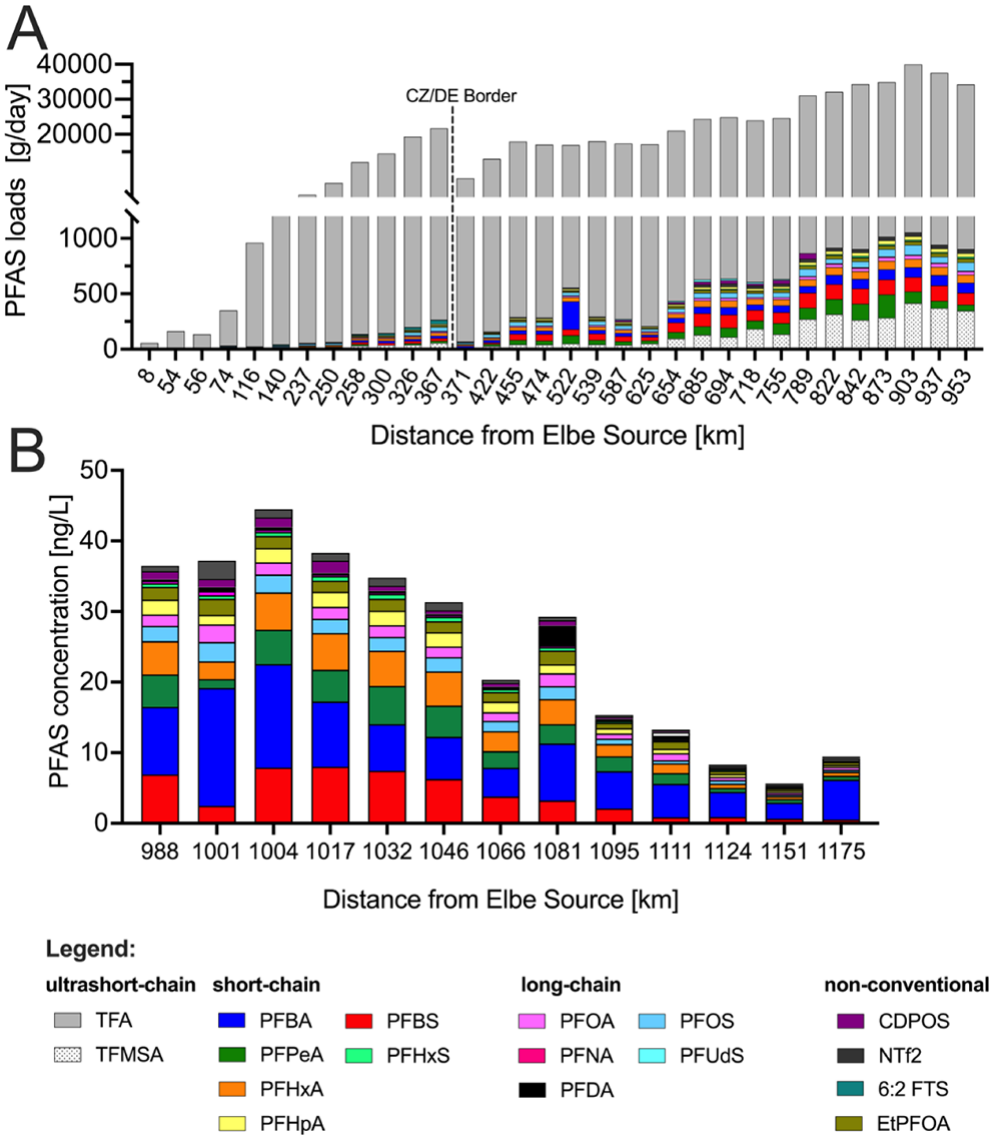
**Longitudinal patterns of PFAS concentrations and mass loads along the Elbe River−North Sea continuum**. (A) Estimated PFAS mass loads (g/day) along the Elbe from Špindlerův Mlýn to Geesthacht, calculated from median PFAS concentrations and corresponding river discharge at each site. (B) Concentrations and distribution of selected PFAS from Hamburg to the North Sea. Note: The 18 PFAS shown were selected based on their frequent detection across freshwater and tidal samples and their representativeness of different PFAS classes (ultrashort-, short-, and long-chain PFAS as well as emerging species). TFA and TFMSA could not be quantified in saline water due to strong matrix effects and was therefore not included in the PFAS quantification for tidal and marine samples in panel B.

#### 3.2.2 Spatial Distribution of PFAS Along the Elbe River

Excluding TFA, PFAS loads in the Czech Elbe section also increased progressively downstream (Figure 2A), indicating cumulative, upstream-dominated inputs. On the German side, PFAS-loads excluding TFA dropped from 270 g/day to 180 g/day between Hrensko (DE, 367 km, 104 m^3^/s) and Schmilka (DE, 371 km, 90 m^3^/s), before increasing again from Schmilka to Geesthacht (DE, 953 km) in line with rising discharge.

However, several deviations from this discharge-driven trend highlight the influence of potential localized sources. A pronounced example is Breitenhagen (DE, 654 km), where short- and long-chain PFAS (without TFA) load reached 456 g/day—more than double the value in nearby Rosslau (DE, 210 g/day at 625 km)—despite only a moderate increase in discharge (to 133 from 109 m^3^/s). This suggests additional inputs in the area, potentially from municipal or industrial wastewater, smaller tributaries, or diffuse sources such as agricultural runoff or sediment remobilization. However, since these values are based on single time-point measurements, temporal variability cannot be excluded and could contribute to observed differences.

Between Breitenhagen (DE, 654 km) and Dömitz (DE, 873 km), PFAS loads increased to 1052 g/day (without TFA), corresponding with the discharge trend (from 133 to 237 m^3^/s). Conversely, between Neu Darchau (DE, 903 km) and Geesthacht (DE, 953 km), PFAS loads declined from 942 to 902 g/day despite stable discharge, suggesting possible retention, degradation, or reduced local input. These patterns underscore the spatial variability of PFAS sources and transport mechanisms within the Elbe system. As this data set is based on a single sampling campaign, temporal variability and underlying source dynamics could not be fully resolved, highlighting the need for seasonal monitoring.

In the absence of discharge data for tidal and marine sites, PFAS concentrations were used instead (Figure 2B). TFA could not be quantified in these samples using our LC or SFC-MS/MS instrument due to strong matrix effects in brackish water. PFAS concentrations generally decreased from Hamburg toward the German Bight (SI_B: Figure S3), indicating progressive dilution and seawater mixing along the river-to-sea continuum. Another driver of decline of PFAS concentration could be the salinity of the water in this area. A different ionic strength in tidal and marine areas could increase the adsorption of certain PFAS to particles and sediments leading to decreasing concentrations, as described by Zhao et al.^16^ and Xiao et al.^35^ In addition to discharge and salinity, dissolved organic carbon (DOC) may also influence PFAS distribution through sorption or complexation effects. DOC concentrations averaged 6.14 ± 0.81 mg/L (SI_A Table S7). No consistent correlation with PFAS concentration was observed, indicating that hydrology rather than DOC controlled spatial patterns, although DOC may facilitate the mobility of short-chain PFAS, because DOC is more likely to interact with short-chain PFAS due to their higher aqueous solubility, whereas long-chain PFAS tend to partition to particles and sediments.^22,36–38^ However, more detailed conclusions about PFAS loads or source identification cannot be drawn without accompanying discharge and salinity data, which were not available in this study.

Across the Elbe river, the overall PFAS load (beyond TFA) was influenced by a few dominant compounds (Figure 2B and SI_B: Figure S1B). Short-chain PFAS—including perfluorobutanoic acid (PFBA, 18%), perfluorobutansulfonic acid (PFBS, 14%), perfluoropentanoic acid (PFPeA, 14%) and perfluorohexanoic acid (PFHxA, 12%)—accounted for the majority of the total load over all samples, while long-chain PFAS such as perfluorooctanesulfonic acid (PFOS, 11%) and perfluorooctanoic acid (PFOA, 5%) also contributed notably. This composition agrees with concentrations of these compounds in previous studies.^9,10,15,16^

Spatially, the relative contribution of long-chain PFAS to the total PFAS load in the respective samples decreased along the transect, from 60% at Kralovstvi (CZ, 54 km) to just 23% in the German Bight (Figure 2A,B). Elevated long-chain PFAS (e.g., PFOS, PFOA) loads in the Czech section may be partially linked to dams near Verdek and Hermanice. These impoundments could reduce flow velocity, promoting sedimentation of hydrophobic compounds like long-chain PFAS. Although sedimentation may decrease water concentrations initially, reduced water exchange and possible resuspension can lead to secondary enrichment in the water column. Similar reservoir effects have been reported by Jin et al.^39^

From the Czech-German border downstream, short-chain PFAS became increasingly dominant. This trend is consistent with the growing use of short-chain alternatives in industrial and consumer applications, as documented by previous studies.^9,12,16^ Overall, the widespread distribution of PFAS and the regional variability in PFAS loads underscore the importance of establishing chemical fingerprints to identify potential sources and also develop better removal technologies.

#### 3.2.3 Chemical Fingerprints of PFAS along the Elbe River

To explore spatial patterns and potential PFAS sources along the Elbe, we applied hierarchical cluster analysis to the concentration data set, segmented into Czech (0−367 km), German freshwater (371−953 km), tidal Elbe (Geesthacht−Cuxhaven, 953−1,065 km), and mouth sections (Cuxhaven−German Bight, 1,066−1,175 km). The three sections were defined a priori based on hydrological boundaries (freshwater, tidal, and estuarine−marine transition) to structure the interpretation of spatial PFAS patterns. We performed a cluster analysis rather than receptor-modeling approaches such as Positive Matrix Factorization (PMF), because PMF requires extensive temporal sampling across multiple seasons to robustly resolve source profiles. Our data set derives from a single, large-scale sampling campaign without temporal replication. Hierarchical clustering is well suited for exploratory analysis of chemical fingerprints and has been successfully applied in other PFAS river studies, e.g., in the Oder River.^40^

In the Czech section (0−367 km), two clusters emerged (Figure 3A):

- Cluster 1: upstream sites such as Špindlerův Mlýn (8 km), with a limited suite of analytes and downstream sites near the Czech/German border showing a broader range of PFAS: perfluorohexansulfonic acid (PFHxS), PFBS, bis­(trifluoromethylsulfonyl)­imide (NTf2), 6:2-fluorotelomersulfonic acid (6:2 FTS), perfluoroethanesulfonic acid (PFEtS), and *N*-ethyl perfluorooctanesulfonamide (N-Et-FOSA).
- Cluster 2 (53−74 km): dominated by long-chain PFAS, e.g., perfluorononanoic acid (PFNA), perfluorodecanoic acid (PFDA), and precursors such as ethyl perfluorooctanoate (EtPFOA) and 5:3 fluorotelomer carboxylic acid (5:3 FTCA). This fingerprint is consistent with inputs from paper and textile industries, which historically used fluorotelomer-based surfactants, and with precursor degradation downstream of paper mills.^11,41,42^

In the German freshwater section (371−953 km), five clusters were identified (Figure 3B).

- Cluster 1 (Schmilka, Dresden): profiles dominated by 6:2 FTS, N-Et-FOSA, PFOS, and perfluorohexanesulfonic acid (PFHxS). This fingerprint aligns with the presence of these compounds—or closely related precursors such as 5:3 FTCA and FTS—in municipal and mixed industrial WWTP effluents, as evidenced by Nguyen et al. (2024).
- Cluster 2 (Riesa−Dommitzsch, 420−540 km): similar to Czech profiles, but with additional co-occurrence of 7H-perfluoroheptanoic acid (HPFHpA) and PFDA, indicating carry-over from upstream sources plus local WWTP inputs.^43,44^

- Cluster 3 (Wittenberg−Werben, 587−789 km) is characterized by capstone B (CDPOS), PFBS, PFPeA, PFHxA, and NTf2. The spatially confined occurrence of CDPOS suggests a localized anthropogenic input in the Bitterfeld−Magdeburg region, which hosts a major chemical manufacturing area.^45^ However, no publicly available records documenting site-specific use or emissions of CDPOS are available. The interpretation is therefore based on spatial co-occurrence patterns rather than confirmed source attribution. Co-occurrence of PFBS and PFHxA is consistent with contributions from municipal wastewater effluents.^17^
- Cluster 4 (Rosslau): low PFAS concentrations without a distinct fingerprint.
- Cluster 5 (Breitenhagen−Geesthacht, 654−953 km): enriched in PFBA, PFPeA, PFHxA, and PFBS, typical of WWTP discharges with poor removal efficiency.^15^ Elevated PFHxA near Hamburg suggests port-related inputs, e.g., AFFF applications^46^ or shipyard activities.

In the tidal Elbe (Geesthacht−Cuxhaven, 954−1,065 km), two clusters were identified (Figure 3C). The site DE_Tide_Elbe_15 (near Hamburg) exhibited the broadest PFAS profile, reflecting harbor industry, shipping activities, and municipal wastewater inputs.

In the Elbe mouth (Cuxhaven−German Bight, 1,066−1,175 km), PFAS fingerprints indicated contributions from industrial port operations and fish-processing facilities, both known estuarine PFAS sources.^47,48^

Across all regions, short-chain PFAS—PFBA, PFBS, PFPeA, PFHxA—were dominant, which is consistent with high mobility, persistence, and poor removal in WWTPs.^15^ Trace levels of PFOA and PFOS in the Elbe are consistent with legacy atmospheric deposition and sediment remobilization.^49^ In addition, PFOA and PFOS may likely stem from ongoing emissions related to historical industrial production, residual use of PFOS and PFOA-containing consumer products, and contaminated sites. NTf2, an emerging PFAS and an anion in ionic liquids, was detected at high frequency along the Elbe River continuum, particularly in the Czech section (258−367 km, Figure 3A) and in central to northern Germany (654−953 km, Figure 3B). NTf_2_-based ionic liquids are widely used in electrochemical systems (e.g., battery electrolytes), metal processing, and specialty chemical manufacturing due to their high chemical stability and broad electrochemical window. The co-occurrence of NTf2 with CDPOS, PFBS, and PFHxA in Location Cluster 3 (Wittenberg−Werben), as resolved by the hierarchical cluster analysis (Figure 3B), indicates a shared industrial fingerprint, consistent with the chemical manufacturing corridor in the Bitterfeld−Magdeburg region, which is a well-documented hotspot for industrial emissions and legacy contamination.^23,50^ Its high polarity and low sorption potential suggest predominantly dissolved transport, yet NTf2 is not included in routine regulatory monitoring.

**Figure 3:**
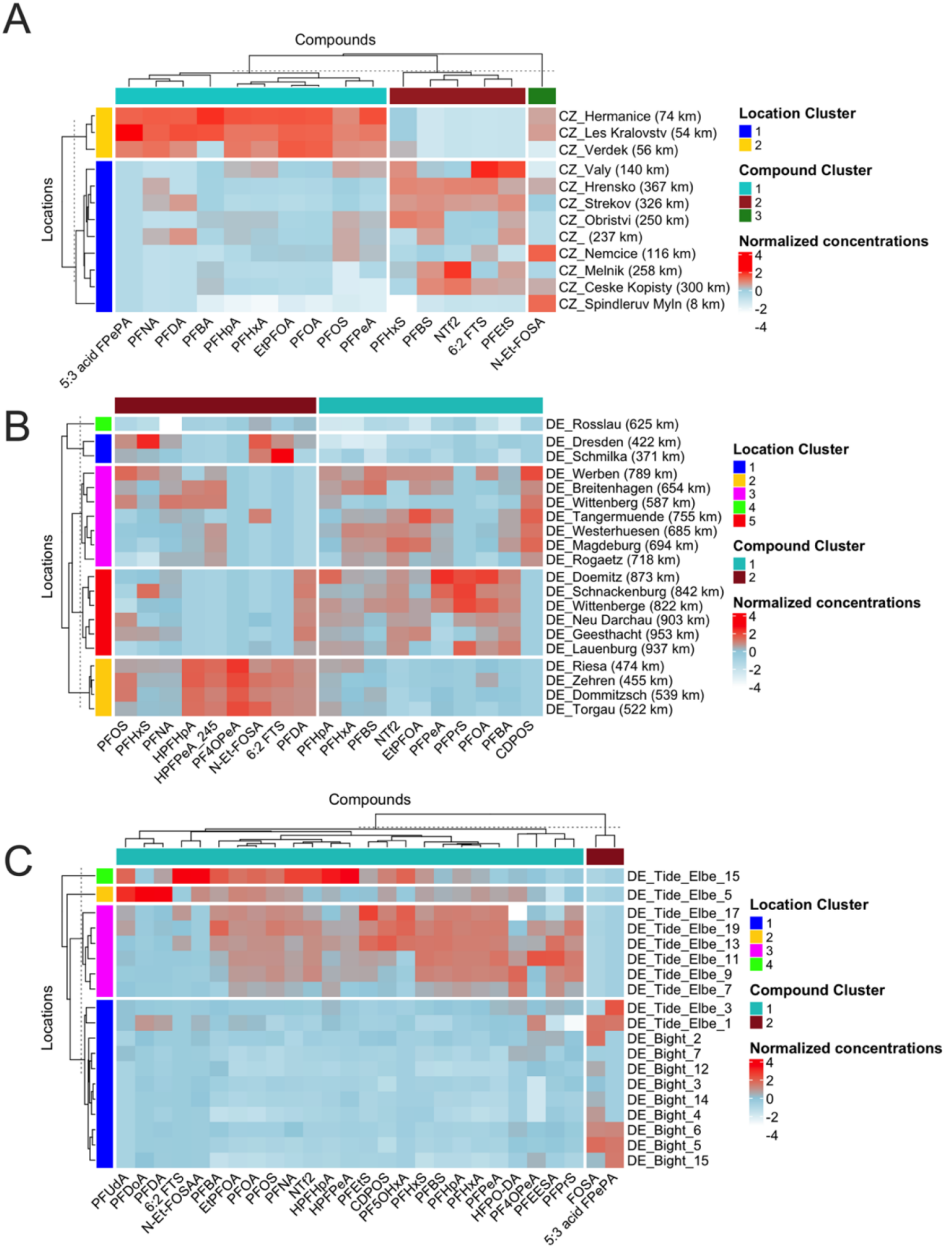
**Clustered heatmaps of normalized PFAS concentrations in three region samples**: (A) Czech regions, (B) German regions, and (C) German tidal and North Sea samples. Locations and compounds are grouped by hierarchical clustering (Ward.D2), revealing distinct spatial and chemical patterns. Colors indicate normalized concentrations.

### 3.3 Contributions of WWTPs and Tributaries to the PFAS Loads in the Elbe River

The spatial variability in PFAS distribution underscores the influence of various contamination sources along the Elbe River. To better understand the impact of these sources, we examined the role of key contributors, including WWTPs and tributaries, in shaping PFAS contamination patterns in the Elbe River system. The 10 sampled WWTPs are located in the Czech Republic (Vrchlabi, Pardubice) and Germany (Dresden, Meissen, Riesa, Torgau, Dessau, Magdeburg, Hamburg, and Hetlingen) (SI_B: Figure S2). Notably, locations with elevated PFAS concentrations (e.g., Hamburg, Magdeburg, Riesa, Dresden, Torgau, Verdek, Kralovstvi, and Hermanice) are situated downstream of WWTPs, suggesting a potential contribution of wastewater effluents to the observed enhanced PFAS concentration.

Given the high number of tributaries, these may have a substantial influence on pollution levels at downstream sites. In particular, the Mulde and Saale rivers are known for their PFAS contamination associated with industrial activities and historical pollution.^10^ Therefore, we further investigated the combined impact of both WWTPs and tributaries on PFAS concentrations in the Elbe River. Thus, the present study provides important knowledge on PFAS inputs into the Elbe river by quantifying PFAS loads and evaluating site-specific PFAS load contributions from WWTPs and tributaries.

#### 3.3.1 Impact of WWTPs on PFAS Profile in the Elbe River

Figure 4A shows PFAS concentrations in ten WWTP effluents along the Elbe River. Without considering ultrashort-chain PFAS (notably TFA), concentrations ranged from 18 ng/L in Vrchlabí (CZ) to 390 ng/L in Pardubice. In contrast, ultrashort-chain PFAS occurred at levels more than 50-fold higher, so excluding them substantially underestimates total effluent concentrations. Dresden showed unusually high short-chain PFAS (230 ng/L) compared to long-chain (29 ng/L), whereas in Riesa long-chain PFAS dominated. Besides input factors, treatment processes may also contribute to lower long-chain concentrations;^51^ for example, chemical phosphorus precipitation applied in Dresden^52^ may promote sorption to sludge, though similar processes likely occur at other sites.

Figure 4B illustrates the contribution of ten WWTP discharges to PFAS levels in the Elbe, expressed as daily loads (g/day). Including ultrashort-chain PFAS increased total loads by one to 2 orders of magnitude. For example, Dresden and Pardubice, which showed the highest loads among the sampled WWTPs, rose from 46 and 13 g/day (excluding TFA) to 185 and 94 g/day (including TFA), respectively. At Hetlingen, loads increased from 9 to 43 g/day. Across all WWTPs, TFA consistently accounted for more than 90% of total PFAS discharge. However, recoveries of TFA were strongly affected by matrix effects (5.3% in influent and 225% in effluent), which introduces uncertainty to elimination estimates and calls for cautious interpretation.

Apart from TFA, short-chain PFAS such as PFBS and PFHxS dominated, consistent with their growing use as replacements for legacy PFAS and their poor removal in conventional treatment. These results align with findings from other European rivers, where urban WWTPs act as major PFAS point sources.^16,53,54^

**Figure 4:**
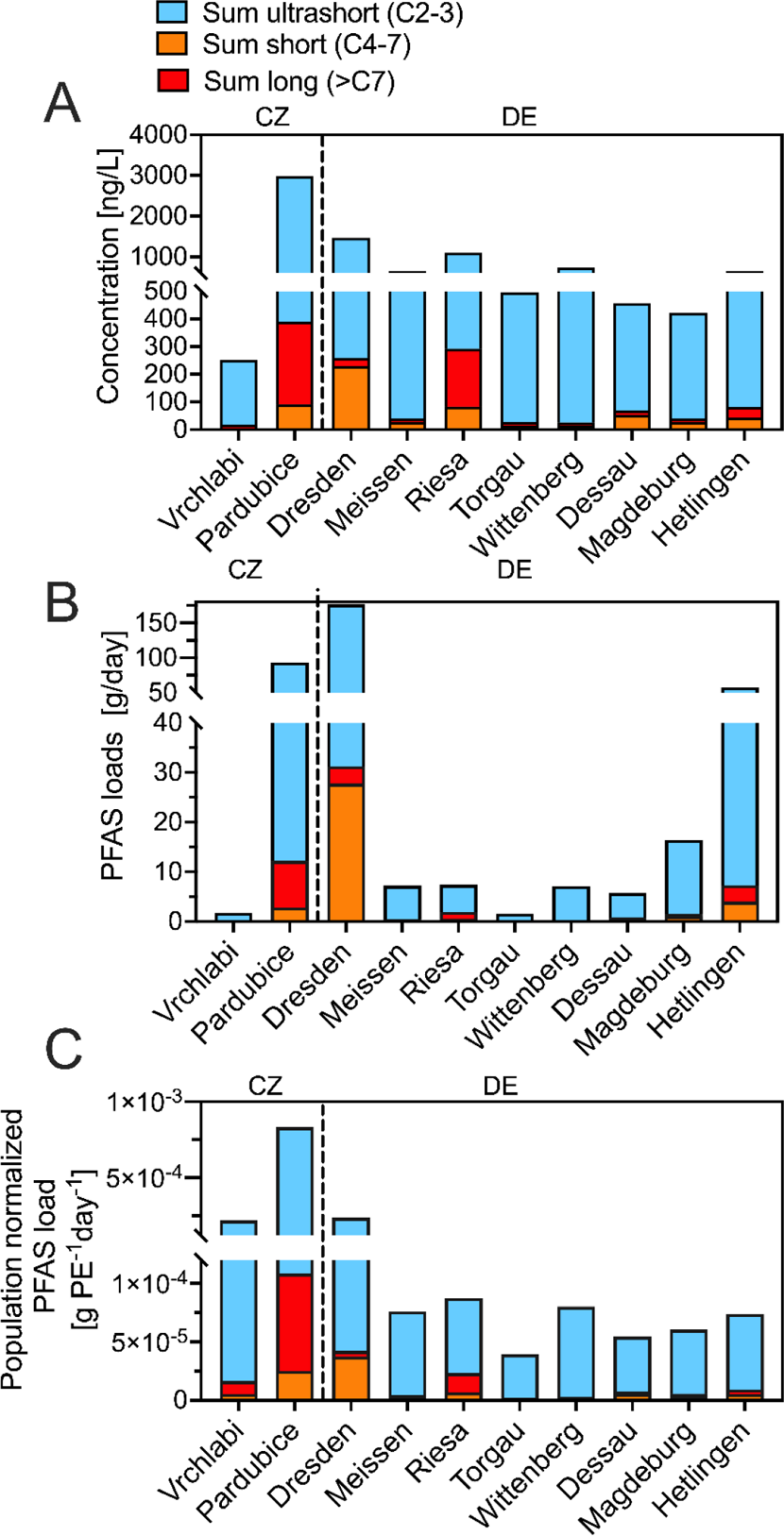
**PFAS concentrations and population-normalized mass loads in WWTP effluents**. (A) Concentrations of ultrashort-, short- and long-chain PFAS in WWTP effluent samples. (B) PFAS loads including ultrashort-chain PFAS based on daily discharge data and determined PFAS concentrations. (C) Population normalized PFAS loads based on population equivalents (PE). Population equivalents include both populations related and industrial discharge of the respective WWTP.

#### 3.3.2 Linking Population Equivalents to PFAS Emissions from WWTPs

To highlight potential sources, PFAS loads were further normalized to population equivalents (PE; one PE corresponds to the average organic load of one person, defined as 60 g biochemical oxygen demand over 5 days (BOD_5_) per day) (Figure 4C). This normalization revealed WWTPs with disproportionately high specific loads not attributable to domestic wastewater alone. Pardubice stood out with markedly elevated values, indicating additional industrial contributions despite industrial inputs already being included in the PE calculation. Similar observations were reported by Krlovic et al., who found that PFAS loads in municipal wastewater often exceed what can be explained by population-based emissions alone.^55^

To further investigate emission patterns beyond TFA, population-normalized loads of selected, frequently detected PFAS representing major substance classes (short-chain, long-chain, emerging PFAS) were visualized using boxplots (Figure 5A). PEs used in this study include both domestic and reported industrial contributions to the respective WWTPs. However, detailed, site-specific information on the type and magnitude of industrial wastewater inputs is not consistently available in public data sets. Therefore, interpretations of elevated PFAS loads at individual plants are based on deviations from population-based scaling and supported by literature rather than on confirmed facility-level source attribution. Short-chain PFAS (e.g., PFBA, PFPeA, PFHxA, PFBS) exhibited relatively narrow ranges (Figure 5A), consistent with predominantly diffuse, population-related emissions.^15,56^ In contrast, some legacy PFAS such as PFOS and PFHxS displayed broader variability and distinct upper outliers, indicating potential additional site-specific inputs. The highest heterogeneity was observed for precursor and emerging PFAS (e.g., 6:2 FTS, NTf2, CDPOS), characterized by pronounced peaks at individual sites and low background levels elsewhere. The variability gradient reflects a transition from diffuse to increasingly localized emission patterns across PFAS classes.

Population-normalized loads of high-variability PFAS revealed locations showing pronounced values across WWTPs (Figure 5B). 6:2 FTS exhibited the strongest peaks at Pardubice (4.82 × 10^−4^ g PE^−1^ d^−1^) and Riesa (5.26 × 10^−4^ g PE^−1^ d^−1^), consistent with previous reports linking elevated 6:2 FTS loads to firefighting foam use and industrial activities.^55^ PFHxS showed a marked maximum at Dessau (3.21 × 10^−5^ g PE^−1^ d^−1^), while PFOS loads were highest at Vrchlabí (1.91 × 10^−5^ g PE^−1^ d^−1^). NTf2 displayed elevated population-normalized loads at Torgau and Vrchlabí, whereas CDPOS population-normalized loads were highest at Riesa and Meißen. The heterogeneous distribution and discrete load peaks support that emissions of these compounds may be driven by localized inputs within specific WWTP catchments rather than diffuse population-related sources.

**Figure 5:**
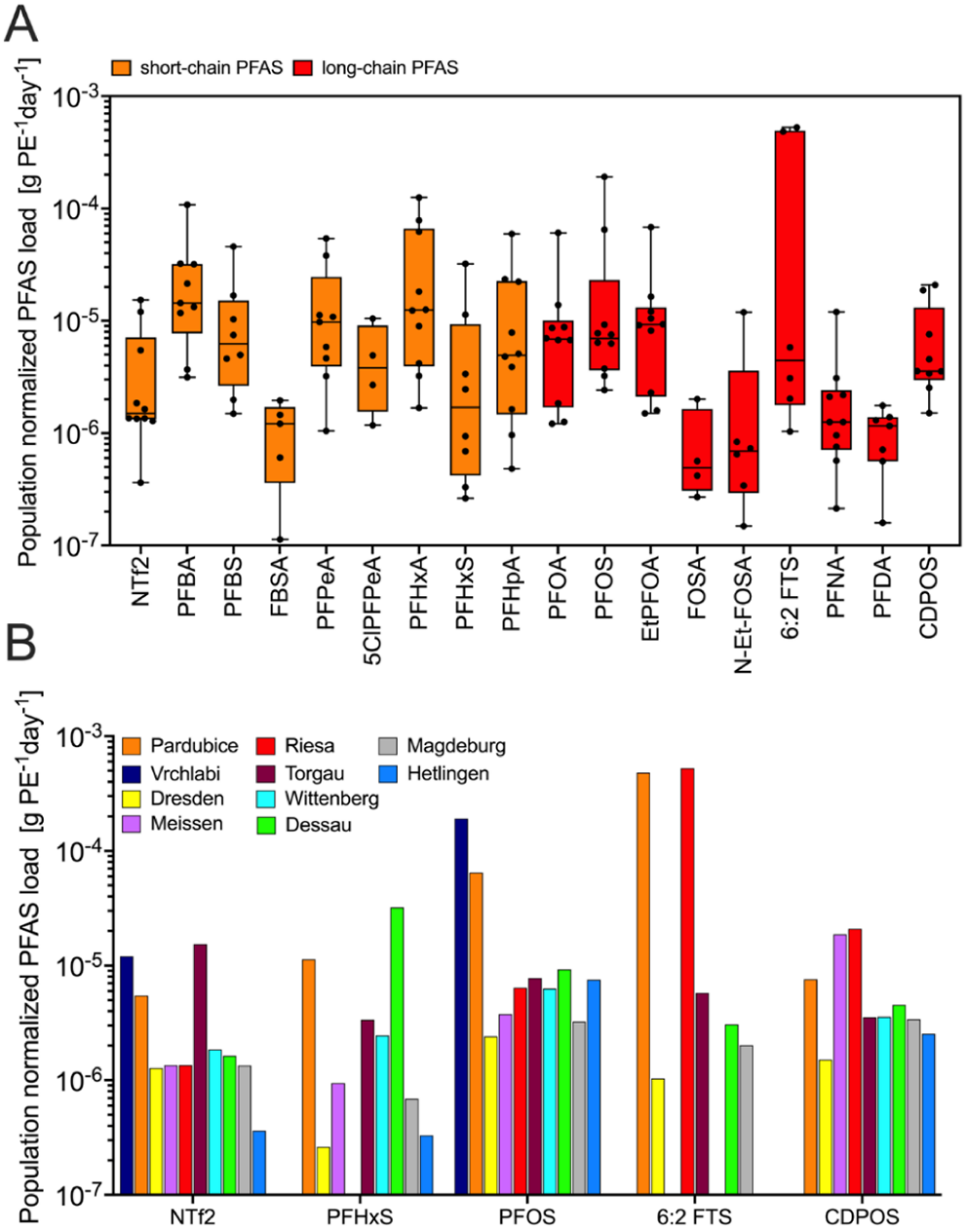
**Population-normalized PFAS loads**. (A) Distribution of population-normalized PFAS mass loads (g PE^−1^ day^−1^) across WWTPs. Boxplots show medians, interquartile ranges, full data ranges, and individual sites. Compounds were selected using a combined data-driven and categorical approach, including frequently detected PFAS with quantifiable emissions and representatives of major substance classes (short-chain, legacy, precursor/telomer-based, emerging PFAS). Variability in normalized loads was used to distinguish diffuse population-related emissions (low variability) from increasingly site-specific or point-source-influenced inputs (high variability). (B) Spatial distribution of population-normalized loads for selected high-variability PFAS (PFHxS, PFOS, 6:2 FTS, NTf2, CDPOS) across individual WWTPs. Selection was based on pronounced variability, sufficient detection frequency, and environmental relevance. Population normalization allows comparison independent of WWTP size and discharge.

#### 3.3.3 Impact of Tributaries on PFAS Profiles in the Elbe River

In addition to municipal WWTPs, tributaries can be important sources of PFAS for the Elbe River. In this study, surface water samples from 16 tributaries were analyzed. Four samples were collected directly at their confluences with the Elbe—Schwarze Elster, Saale, Havel, and Mulde—thus capturing the cumulative impact of upstream sources across each catchment. In 13 of the 16 tributaries, total PFAS concentrations (sum of short- and long-chain compounds, excluding TFA) remained below 30 ng/L, similar to levels observed at most Elbe mainstream sites. Median TFA concentrations in Elbe tributaries was 837 ng/L ranging from 500 ng/L in Ploucnice (CZ) to 1436 ng/L in Weisseritz (DE), comparable to TFA concentrations in the Elbe river. Moreover, substantially elevated total PFAS concentrations (when excluding TFA) were observed in the Saale (40.1 ng/L), Mulde (71.8 ng/L), and especially the Bilina (233 ng/L), indicating that local sources such as industrial activity, wastewater discharges, and population density play a major role in PFAS contamination within these catchments. Determined total PFAS concentrations (without TFA) are consistent with recent national monitoring data compiled in the German UBA, which also identified elevated PFAS concentrations in tributaries such as the Saale, Mulde, and Havel, while concentrations in the Elbe generally remained below 30 ng/L.^57^

To assess their contribution to the Elbe’s PFAS burden, loads were calculated based on measured concentrations and discharge values (Figure 6A). Although the Bilina had the highest concentration (1,527 ng/L), its contribution to total PFAS load was relatively low (142 g/day) due to limited flow (1.59 m^3^/s). In contrast, the Vltava (6,089 g/day; 59.8 m^3^/s), Saale (2,340 g/day; 45.7 m^3^/s), Havel (3,143 g/day; 40.2 m^3^/s), and Mulde (1,700 g/day; 12.7 m^3^/s) contributed substantially higher PFAS loads. Together, the major tributaries account for approximately 67% (158 m^3^/s) of the Elbe’s discharge at Geesthacht (240 m^3^/s), the last nontidal cross-section of the river,^29,58^ highlighting their dominant hydrological role in shaping downstream PFAS fluxes. However, this comparison is limited by the assumption of conservative transport and temporal alignment of discharge and concentration data, as in-river retention, remobilization, and additional downstream sources may alter PFAS loads between tributary confluences and the Geesthacht cross-section. Despite these limitations, this comparison offers a first-order, system-scale framework to rank major tributaries by their relative contribution to the Elbe’s PFAS mass balance. The Rhine, in contrast, transports several kilograms of PFAS per day (4−6 kg/day) without TFA), despite similar concentrations (20−40 ng/L), due to its significantly higher discharge^59^ (2,300 m^3^/s) and extensive industrial catchment.^60^

**Figure 6:**
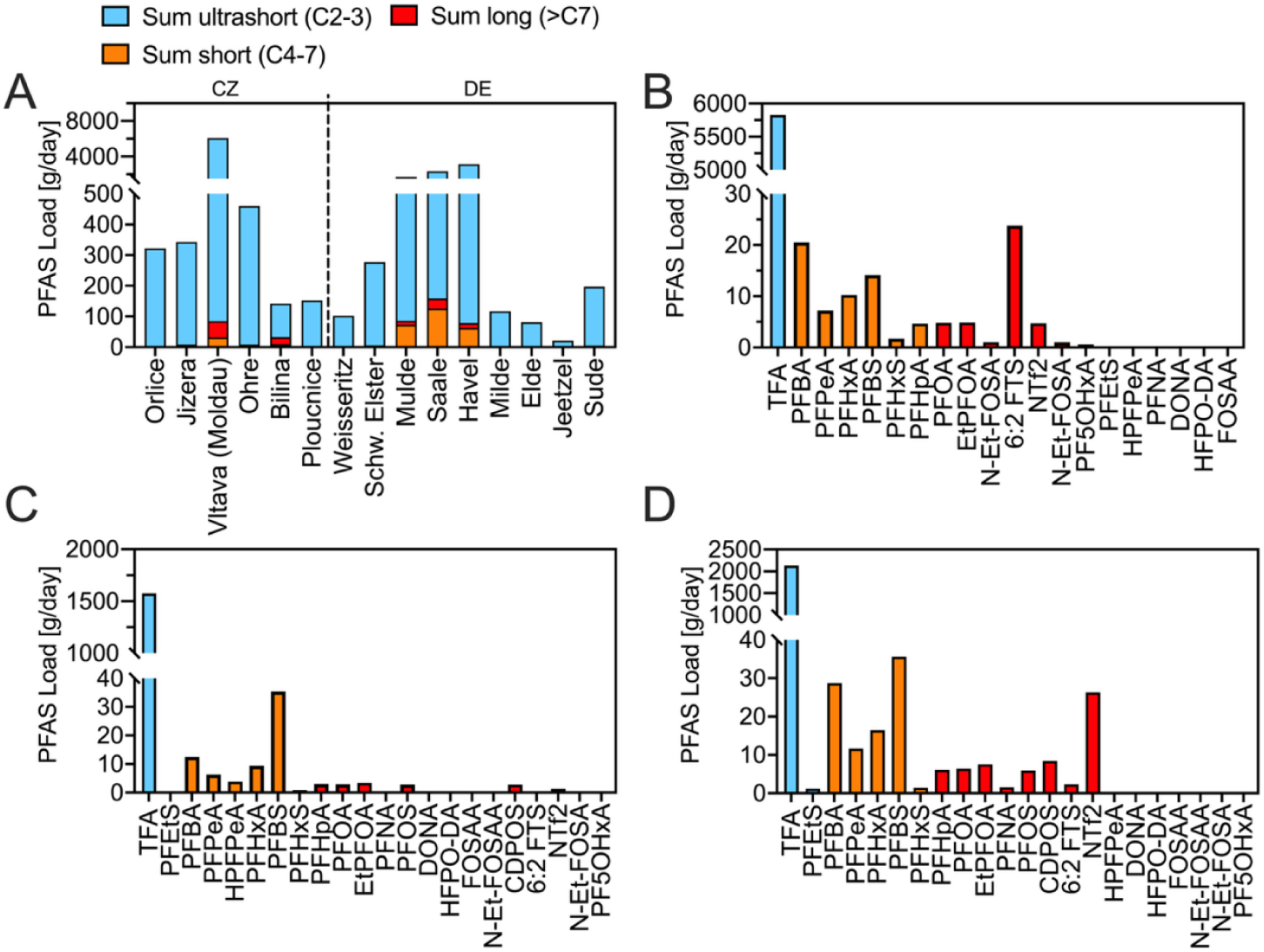
**PFAS composition across 16 tributaries and estuaries of the Elbe River spanning from the Czech Republic to Germany**. (A) Distribution of short- and long-chain PFAS loads in 16 tributaries of the Elbe River. PFAS concentration profiles in the highly contaminated (B) Vltava, (C) Mulde, and (D) Saale rivers.

In addition, PFAS profiles differed regionally. Excluding TFA, the Vltava’s load was slightly dominated by long-chain PFAS (62%), whereas the Saale, Havel, and Mulde were primarily characterized by short-chain PFAS. These differences likely reflect variations in local industrial activities, land use, and emission sources. The elevated PFAS loads observed here are consistent with previous findings; for instance, Göckener et al. reported high concentrations of short-chain PFAS in the Saale and Mulde rivers, while the Havel was also identified as a substantial source of PFAS—predominantly long-chain compounds.^9,10^ The high PFAS loads from Saale and Mulde rivers are consistent with historical and contemporary contamination from chemical production sites in regions such as Bitterfeld-Wolfen.^9,13^

Further examination of PFAS profiles (Figure 6B) reveals that the Vltava’s load is dominated by 6:2 FTS, PFHxA, PFBS, PFPeA, and PFBA—all of which were found at notable concentrations in the Elbe, indicating a strong input of these PFAS. Similarly, the Mulde’s PFAS profile is (Figure 6C) shaped primarily by PFBS, PFBA, HPFPeA, PFPeA, and PFHxA, in agreement with previous literature.^10^ This could partly explain their widespread presence in the Location Cluster 3 (Figure 3B), where the Mulde joins the Elbe, though additional sources cannot be excluded. Furthermore, CDPOS was detected in the Mulde tributary with a mass load of 2.8 g/day (Figure 6C). The Saale—showing the second-highest overall PFAS load—showed a 3-fold higher CDPOS mass load (8.4 g/day, Figure 6D), with elevated concentrations predominantly observed at downstream Elbe sites between Wittenberg (3.8 ng/L) and Werben (2.7 ng/L) (Figure 3B). The Bílina River (CZ) was also identified as a source of CDPOS to the Elbe, with a measured concentration of 69 ng/L. However, due to its comparatively low discharge (1.58 m^3^/s), its overall contribution to the CDPOS load in the Elbe is limited. The spatial coincidence of these elevated loads with the respective confluence zones indicates that the Saale and the Mulde catchments likely represent major input pathways for CDPOS into the Elbe. Notably, CDPOS was not detected, or occurred only at substantially lower levels (<1 ng/L), at other major industrial and port regions along the Elbe (e.g., Dresden−Riesa, Magdeburg, and Hamburg), suggesting a spatially confined source pattern rather than generalized industrial emissions. This selective distribution is consistent with an anthropogenic origin associated with specific industrial or infrastructure-related applications, such as the use or handling of fluorinated surfactants or specialty chemicals. CDPOS is a polyfluorinated surfactant that has been reported as a component of certain aqueous film-forming foams (AFFF) and related fluorochemical formulations, which are recognized as potential environmental input pathways for PFAS.^2,61,62^ However, without compound-specific information on emissions, usage, or discharge pathways, and given the reliance on a single grab sampling campaign, the proposed source linkage remains preliminary and cannot be interpreted as conclusive source attribution. The detection of NTf2 in the Saale tributary with 26 g/day is consistent with the influence of industrial activities within the central German chemical manufacturing region on PFAS inputs to the Elbe.

Thus, including TFA in such assessments would substantially increase total PFAS monitoring values, highlighting the relevance of TFA monitoring in surface water. The Danube also shows elevated PFAS loads, with peak discharges of 6−9.5 kg/day near urban and industrial centers such as Budapest and Ruse.^63^ Thus, while the Elbe is less affected by diffuse PFAS inputs than Rhine or Danube, localized contributions from major tributaries make it a regionally substantial recipient of PFAS contamination. However, as our data set represents a single low-flow campaign in summer 2023, additional monitoring across seasons and hydrological conditions is required to capture temporal variability of PFAS loads.

### 3.4 Environmental Risk Assessment

RQs showed that most PFAS remained below the screening threshold of RQ = 0.1 and are therefore of limited immediate ecotoxicological concern. Elevated RQs were restricted to a small subset of compounds (Figure 7).

PFOS displayed the highest risk levels with median RQ of 4.98 and a maximum of even 82 detected in Bilina indicating consistent ecological relevance across sampling sites.

PFHxS and several long-chain PFCAs (PFNA, PFDA, PFOA, EtPFOA) showed moderate but locally increased RQs (0.1 < RQ < 1), partially exceeding the RQ = 1 threshold. In contrast, precursor and emerging PFAS such as 6:2 FTS, NTf_2_, and CDPOS exhibited lower median RQs but distinct upper values, suggesting localized exposure rather than system-wide risk.

While most short-chain PFAS such as PFBA, PFPeA, and PFBS showed RQs far below 1 (SI_A, Table S10), indicating no acute ecological risk under current thresholds. TFA (RQ < 0.1), while dominating PFAS loads, exhibited RQs well below available PNEC values (110 μg/L, see SI_A Table S9). Although not acutely toxic, its massive fluxes to the North Sea emphasize the need for future regulatory consideration. However, frameworks for regulatory relevance of potential mixture effects of diverse PFAS should be established to evaluate mixture toxicity in the future.^64–66^

It is needed to mention that interpretation of these results is constrained by PNEC availability and quality. While legacy PFAS are supported by experimentally derived or regulatory thresholds, many emerging PFAS rely on predicted screening-level PNECs compiled from the NORMAN database, often based on modeled toxicity estimates. Accordingly, RQs for these compounds should be regarded as indicative prioritization metrics within a monitoring context rather than definitive ecological risk evaluations.

**Figure 7:**
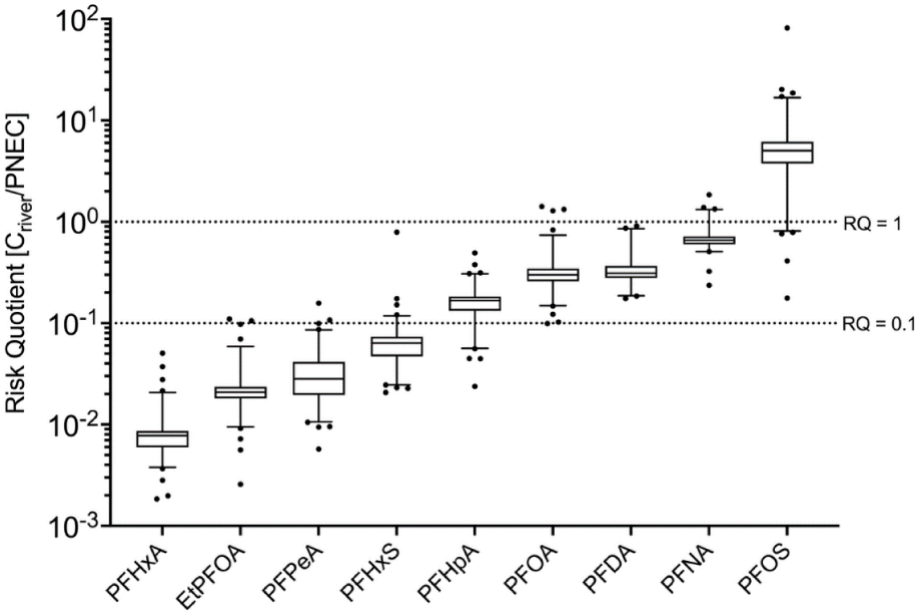
**Distribution of RQs for PFAS exceeding screening thresholds (RQ > 0.1) across all river water samples including tributaries (** ***n*** **= 88).** Boxplots display medians, interquartile ranges, and full data ranges (whiskers: min−max). Compounds are ordered by increasing median RQ from left to right. Horizontal reference lines indicate RQ = 0.1 and RQ = 1, representing moderate and high ecological risk levels, respectively. PNEC data were obtained for freshwater from NORMAN Ecotoxicology Database. Details on PNEC values and individual references are provided in SI_A Table S9. RQs of all compounds and samples are provided in Table S10 in SI_A.

## 4 Conclusions

This study provides a comprehensive data set on PFAS occurrence and transport along the Elbe, covering 38 detected compounds from headwaters to the North Sea. A central finding is the overwhelming dominance of TFA, which contributed >96% to the total PFAS load but is currently excluded from routine regulatory monitoring. Its omission therefore leads to a systematic underestimation of PFAS fluxes in European rivers. By applying compound-specific load calculations and cluster analysis, we demonstrated that the Elbe is shaped by both diffuse inputs (e.g., atmospheric deposition, precursor degradation) and regional point sources (e.g., WWTP effluents, industrial discharges, historical contamination). These chemical fingerprints underline the heterogeneity of PFAS contamination and provide an evidence base for source-oriented management. Importantly, the detection of CDPOS—a compound rarely reported in surface waters—highlights the presence of industrially derived, nonroutinely monitored PFAS that may locally shape river fingerprints. Although risk quotients were below 1 for the most of the detected PFAS with available PNEC/toxicity data, this does not imply environmental safety, as chronic PNECs, mixture toxicity data, and precursor transformation products are largely missing, leaving a major uncertainty for risk assessment.

Building on these findings, river-basin monitoring frameworks should be adapted to better capture highly mobile and regionally relevant PFAS. This includes extending routine target lists beyond legacy compounds to cover ultrashort-chain and selected emerging PFAS (e.g., TFA, NTf_2_, CDPOS), combining concentration measurements with mass-load calculations, and systematically sampling at major tributary confluences and hydrological transition zones. Such a design would improve the identification of source-enriched inputs, support targeted mitigation at the catchment scale, and provide a more robust basis for revising EU monitoring programs and water quality standards.

## Supplementary Material





## Notes

Excel file (**SI_A**): contains the sampling locations, PFAS targets, method performance, water chemistry, a complete data set of quantified PFAS concentrations across all sampling locations and matrices included in the study, as well as PNECs and RQ data (XLSX)
PDF document (**SI_B**): provides detailed descriptions of analytical methods (**Tables S1−S2**), results of principal component and hierarchical cluster analyses (**Figure S1**, **Tables S3−S4**), and supplementary figures and tables illustrating spatial PFAS distribution patterns (**Figures S2−S3, Table S5**) (PDF)

## Abbreviations

ACN: acetonitrile
AFFF: aqueous film-forming foams
BEH: ethylene bridged hybrid
CZ: Czech Republic
CDPOS: Capstone B, 6:2 fluorotelomer sulfonamido propyl betaine
DE: Germany
DiPAPs: polyfluorinated dialkyl phosphates
ESI: electrospray ionization
EtPFOA: ethyl perfluorooctanoate
EFF: effluent
INF: influent
LC-MS/MS: liquid chromatography−tandem mass spectrometry
MeOH: methanol
MRM: multiple reaction monitoring
NTf2: bis­(trifluoromethylsulfonyl)­imide
PNEC: predicted no-effect concentration
PFAS: per- and polyfluoroalkyl substances
PFBA: perfluorobutanoic acid
PFBS: perfluorobutanesulfonic acid
PFPeA: perfluoropentanoic acid
PFHxA: perfluorohexanoic acid
PFHpA: perfluoroheptanoic acid
PFHxS: perfluorohexanesulfonic acid
PFOS: perfluorooctanesulfonic acid
PFOA: perfluorooctanoic acid
RP-UPLC-MS/MS: reversed-phase UPLC−tandem mass spectrometry
SFC: supercritical fluid chromatography
SFC-MS/MS: SFC−tandem mass spectrometry
SI: Supporting Information
TFA: trifluoroacetic acid
TQS: triple quadrupole system (Waters TQS)
UPLC: ultraperformance liquid chromatography
WWTPs: wastewater treatment plants
