# Supplementary material for: Beyond Legacy PFAS: Dominant Role of Ultrashort-Chain and Emerging PFAS in the Elbe River−Sea Continuum
Source: ACS Environ Au. 2026 Mar 18;6(3):421–34. doi: 10.1021/acsenvironau.5c00219 (PMC13195463; doi:10.1021/acsenvironau.5c00219)
Supplement: Supplementary file 2 [file vg5c00219_si_002.pdf]

Supporting information_B

Beyond Legacy PFAS: Dominant Role of Ultrashort-Chain and Emerging PFAS in the Elbe River–Sea Continuum

Anne Röhrig[a], Nico Grasse[a], Martin Krauss[b], Dana Bücher[a], Werner Brack[b,d], Norbert Kamjunke[e], Anna Matousu[f], Tina Sanders[g], Ingeborg Bussmann[h], Eric P. Achterberg[i], Thorsten Reemtsma[a,c], Qiuguo Fu[a*]

[a]Department of Environmental Analytical Chemistry, Helmholtz-Centre for Environmental Research – UFZ, Permoserstrasse 15, 04318 Leipzig, Germany.

[b]Department of Exposure Science, Helmholtz-Centre for Environmental Research – UFZ, Permoserstrasse 15, 04318 Leipzig, Germany.

[c]Institute for Analytical Chemistry, University of Leipzig, Linnestrasse 3, 04103 Leipzig, Germany.

[d]Department of Evolutionary Ecology and Environmental Toxicology, Faculty of Biological Sciences, Goethe University Frankfurt, Max-von-Laue-Strasse. 13 60438 Frankfurt am Main, Germany.

[e]Department of River Ecology, Helmholtz Centre for Environmental Research − UFZ, Magdeburg 39114, Germany.

[f]Biology Centre, Czech Academy of Sciences, Institute of Hydrobiology, Ceské Budejovice 370 05, Czech Republic

[g]Institute of Carbon Cycles, Helmholtz Centre Hereon, Geesthacht 21502, Germany

Ingeborg Bussmann − Department of Shelf Sea System Ecology, Alfred-Wegener-Institut, Helmholtz Zentrum für Polar- und Meeresforschung, Helgoland 27498, Germany

[h]Department of Shelf Sea System Ecology, Alfred-Wegener-Institut, Helmholtz Zentrum für Polar- und Meeresforschung, Helgoland 27498, Germany.

[i]GEOMAR, Helmholtz Centre for Ocean Research Kiel, Kiel 24148, Germany

***Corresponding author**: Qiuguo Fu, Email: qiuguo.fu@ufz.de

Number of Figures: 4

Number of Tables: 3

Number of pages: 12

Table of contents

1. **CHROMATOGRAPHIC PARAMETERS** 4
2. **DATA ANALYSIS** 5
  2.1 Multivariate Analysis 5
  2.2 Cluster Analysis 5
  2.3 Principal Component Analysis (PCA) 5
5. **GIS MAPPING** 7
6. **CONCENTRATIONS OF PFAS IN TIDAL AND SEA WATER SAMPLES** 8
7. **PCA ANALYSIS OF PFAS CONCENTRATION PROFILES** 9

Figures

Figure S1: (**A**) Distribution of concentrations of distinct PFAS along the Elbe River. TFA was not quantified in tidal and marine water samples from Hamburg (998 km) on due to incompatibility with SFC-MS/MS instrumentation. (**B**) Relative contributions of concentrations of relevant PFAS to overall samples in the Elbe River in Germany without the contribution of TFA. 6
Figure S2: Locations of Waste Water Treatment Plants (WWTPs), Elbe tributaries and locations with PFAS concentrations higher than 30 ng/L. 7
Figure S3: Sum of short-and long-chain PFAS concentrations depending on the sampling distance from Hamburg 8
Figure S4: Principal Component Analysis (PCA) of PFAS concentration profiles across compounds 9

Tables

Table S1: Eluent composition RP-LC....4
Table S2: Eluent composition SFC 4
Table S3: Concentrations of short- and long-chain PFAS in tidal and German bight samples....10

1. Chromatographic Parameters

Table S1: Eluent composition RP-LC

Eluent A	95% MilliQ + 5% MeOH + 2mM NH4oAc	
Eluent B	75% MeOH + 20% ACN + 5% MilliQ + 2mM NH4oAc	
Time (min)	% A	% B
0	90	10
1.5	90	10
4.5	35	65
8.25	20	80
8.26	0.1	99.9
11	0.1	99.9
11.01	90	10
15	90	10

Table S2: Eluent composition SFC

Eluent A	CO2	
Eluent B positive mode	95%MeOH + 5% Ultra pure water + 10mM NH4Fa	
Eluent B negative mode	95%MeOH + 5% Ultra pure water + 10mM NH4OH	
Make-up flow eluent	90%MeOH + 10% Ultra pure water + 0.1% formic acid	
Make-up flow	0.15 ml/min	
Time (min)	% A	% B
0	99	1
1	99	1
13	50	50
16	50	50
16.5	99	1
18	99	1

2. Data Analysis

2.1 Multivariate Analysis

To explore spatial and chemical trends in PFAS occurrence along the Elbe River, multivariate analyses were performed using R (v4.2.0). A matrix of log-transformed and Z-scaled PFAS concentrations (maximum values per site) was used as input. Concentrations below the limit of detection (LOD) or limit of quantification (LOQ) were set to zero. The dataset included riverine sampling sites as rows and individual PFAS compounds as columns.

2.2 Cluster Analysis

Hierarchical clustering was conducted using Ward's method (ward.D2) with Euclidean distance. The optimal number of clusters for both sites and compounds was identified using the silhouette width criterion, evaluated across cluster numbers from 2 to 10. Clusters were assigned using the cutree function. Results were visualized with the ComplexHeatmap package, employing a white-to-red color gradient to indicate normalized PFAS concentrations. Custom color schemes were applied to annotate both site and compound clusters.

2.3 Principal Component Analysis (PCA)

To support cluster-based groupings, separate PCAs were conducted on site-wise and compound-wise datasets using the prcomp function. The first two principal components were plotted with ggplot2, including 95 % confidence ellipses to highlight cluster separation. Variance explained by each dimension (Dim1 and Dim2) was reported in the plots. Results are plottet in **Figure S2**.

3. Median Concentrations of PFAS along the Elbe river

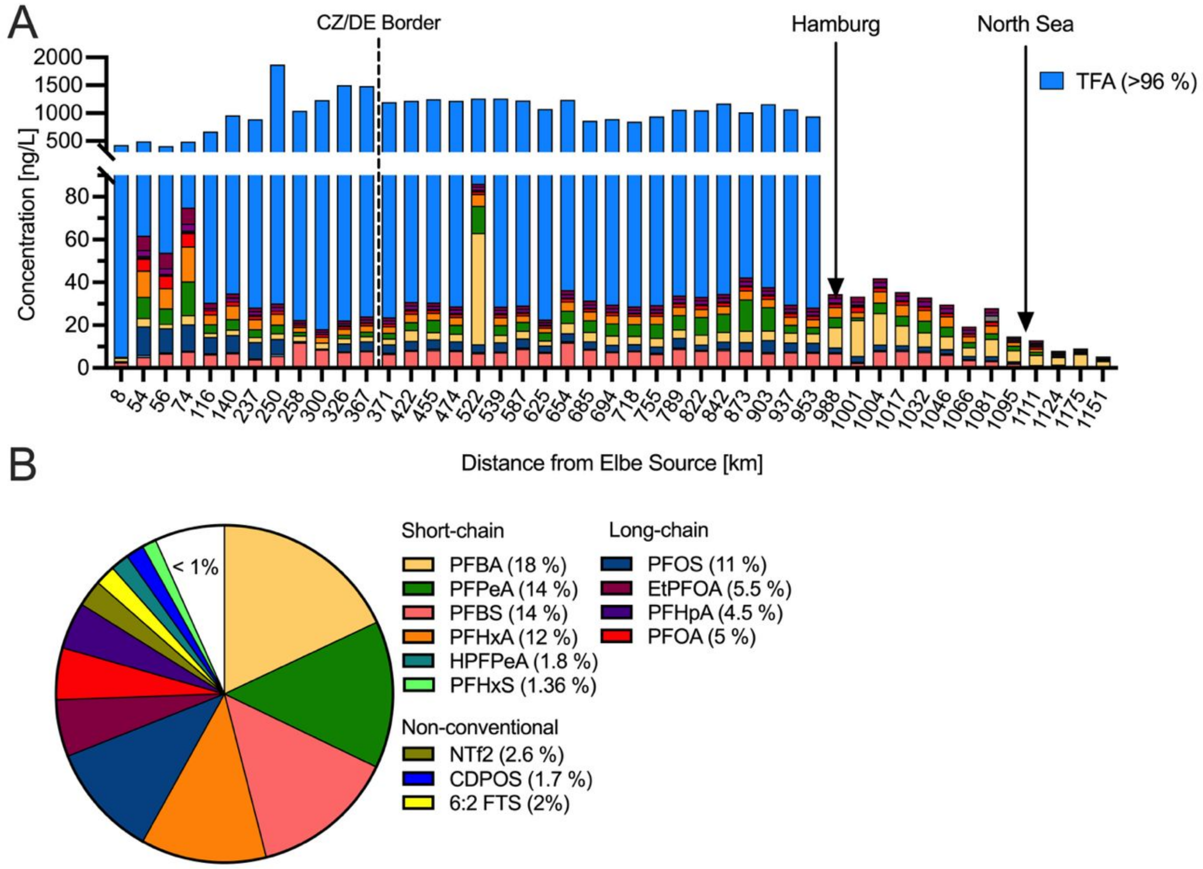

Figure S1: (**A**) Distribution of concentrations of distinct PFAS along the Elbe River. TFA was not quantified in tidal and marine water samples from Hamburg (998 km) on due to incompatibility with SFC-MS/MS instrumentation. (**B**) Relative contributions of concentrations of relevant PFAS to overall samples in the Elbe River in Germany without the contribution of TFA.

4. Locations of Samling Points

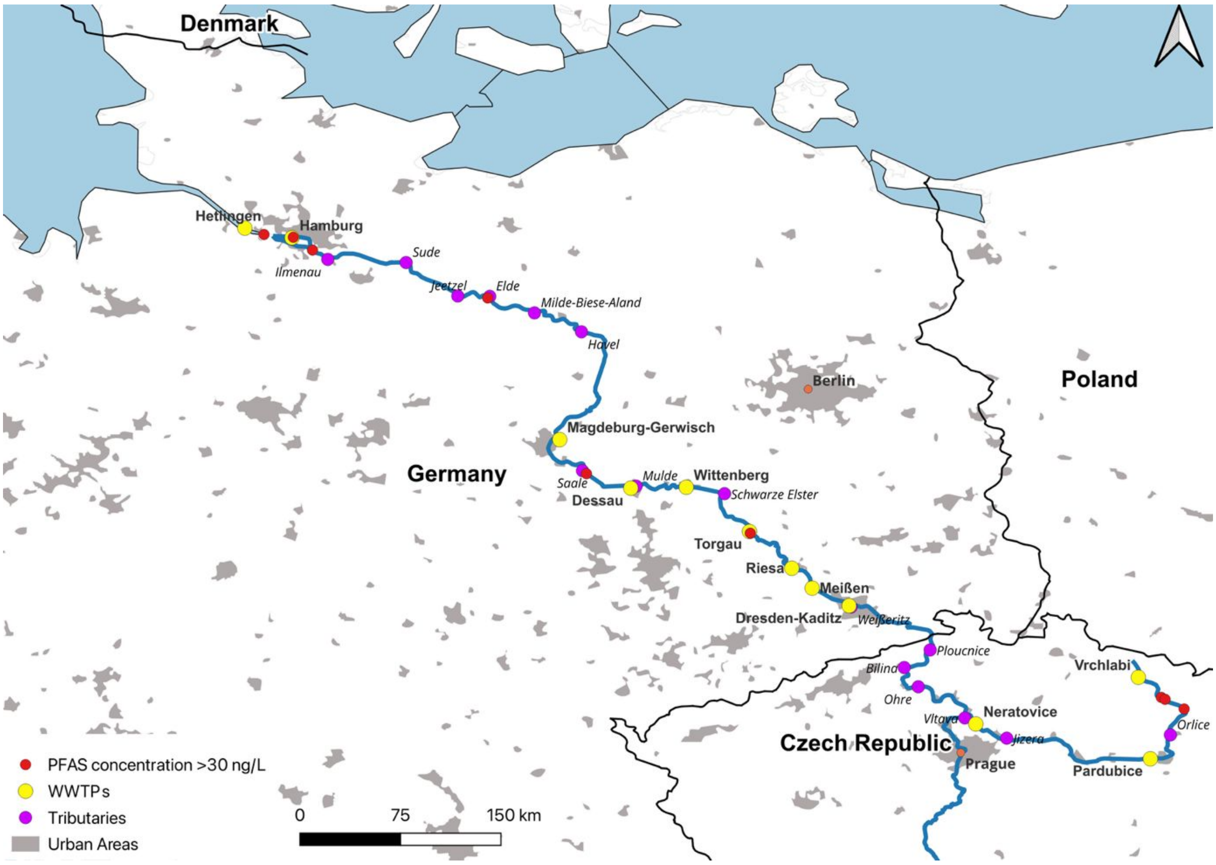

Figure S2: Locations of Waste Water Treatment Plants (WWTPs), Elbe tributaries and locations with PFAS concentrations higher than 30 ng/L.

5. GIS Mapping

Sampling site coordinates (latitude and longitude, WGS 84) were imported into QGIS version 3.40.5 “Bratislava” as a Delimited Text Layer and converted to a point shapefile. All layers were reprojected to EPSG:4326. The Natural Earth Quick Start Kit (v3) served as the base map, including vector layers for land, water bodies, coastlines, and urban areas. Urban regions were emphasized using the “urban_areas” polygon layer with a semi-transparent gray fill. Base layers (land, rivers, and coastlines) were displayed at 1:50 m scale to balance detail and clarity. Graphic properties such as color palettes, label fonts, and layer order were adjusted via the Layer Styling panel to ensure effective cartographic representation.

6. Concentrations of PFAS in tidal and sea water samples

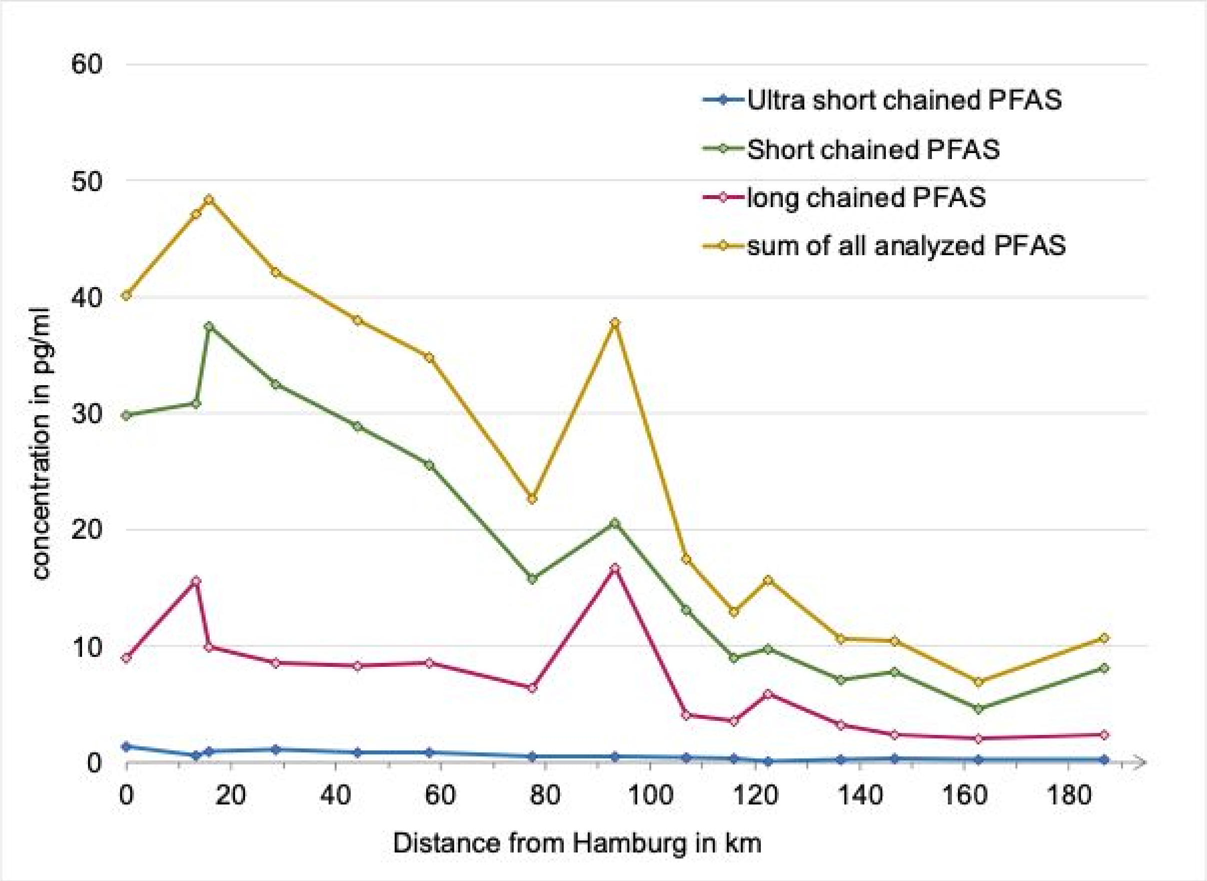

Figure S3: Sum of short-and long-chain PFAS concentrations depending on the sampling distance from Hamburg

7. PCA Analysis of PFAS concentration Profiles along the Elbe river

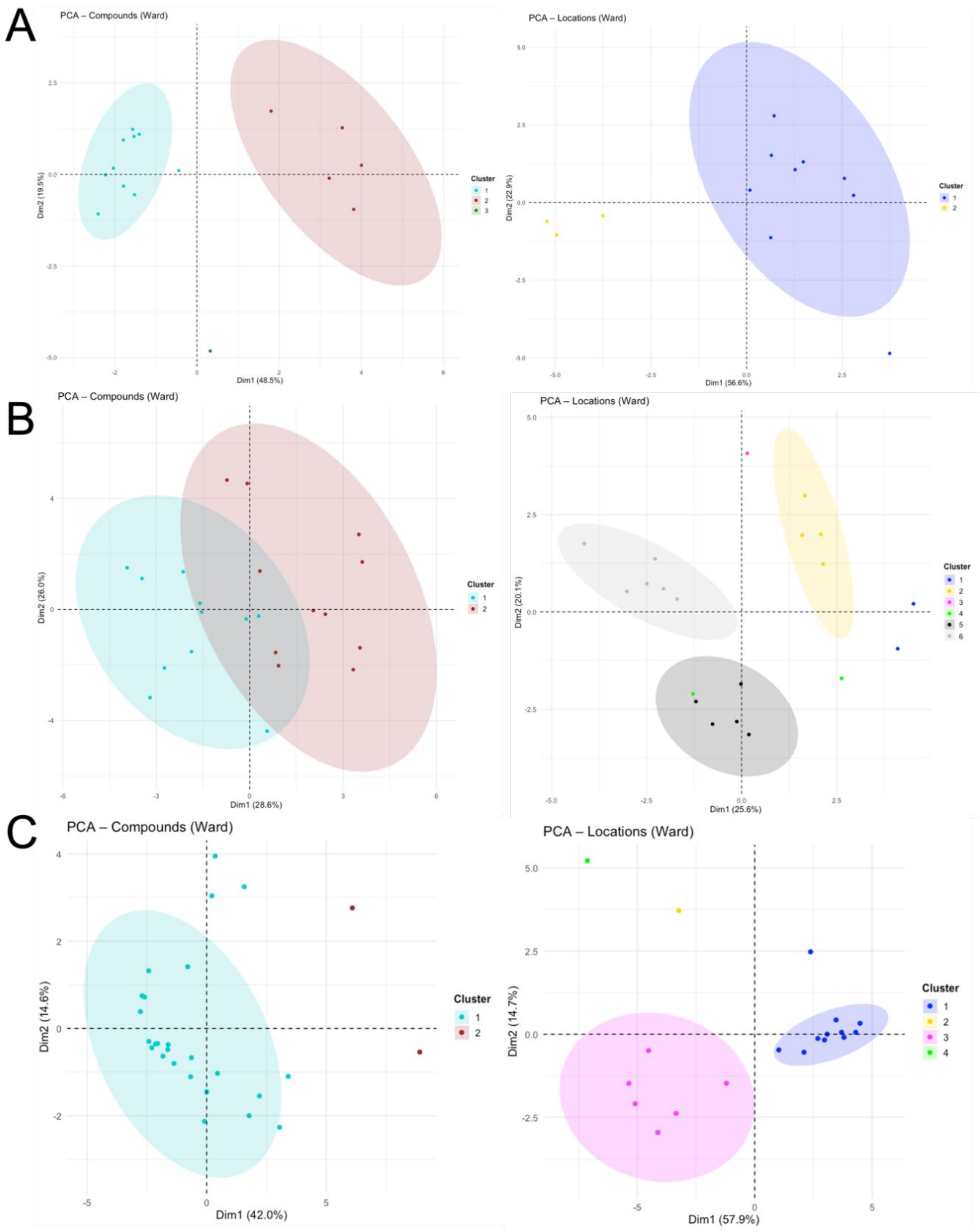

Figure S4: Principal Component Analysis (PCA) of PFAS concentration profiles across compounds (left) and sampling locations (right), (**A**) in Czech Republic, (**B**) German Sites, (**C**) Tidal areas and Bight. Clusters were determined using hierarchical Ward clustering and visualized in PCA space. Ellipses represent 95% confidence intervals based on the multivariate normal distribution. PCA was conducted on log-transformed and z-scaled data.

The analysis reveals distinct grouping patterns suggesting similarities in compound occurrence and site-specific contamination profiles.

Table S3: Concentrations of short- and long-chain PFAS in tidal and German bight samples.

Sample	Sum short-chain PFAS [ng/L]	Sum long-chain PFAS [ng/L]
GB02	9.812	4.411
GB03	7.8525	3.292
GB04	4.846	2.205
GB05	7.352	3.066
GB08	9.007	3.597
GB09	7.739	2.363
GB12	8.079	2.355
GB14	7.128	3.24
GB15	4.641	2.038
**Average Bight Samples**	**7.381**	**2.952**
**Standard Deviation**	**22%%**	**25%**
ET01	9.714	5.905
ET03	13.074	4.063
ET05	20.606	16.727
ET07	15.744	6.397
ET09	25.53	8.515
ET11	28.912	8.251
ET13	32.529	8.556
ET15	30.885	15.604
ET17	29.868	8.93
ET19	37.525	9.917

**Average Tidal Samples**	**24.439**	**9.293**
**Standard Deviation**	**36%**	**41%**
